# Generation of a planar direct-current glow discharge in atmospheric pressure air using rod array electrode

**DOI:** 10.1038/s41598-017-03007-1

**Published:** 2017-06-01

**Authors:** Xuechen Li, Panpan Zhang, Pengying Jia, Jingdi Chu, Junying Chen

**Affiliations:** 1 College of Physics Science & Technology, Hebei University, Baoding, 071002 China; 2 Key Laboratory of Photo-Electronics Information Materials of Hebei Province, Baoding, 071002 China

## Abstract

Scaling up atmospheric pressure glow discharge to large volume is desirable for low-temperature plasma applications. In this paper, an approach to generate a glow discharge in a planar shape with a fairly large volume is proposed in atmospheric pressure air through utilizing a direct-current excited rod array electrode. The planar discharge with a wide gap originates from three discrete discharges with a narrow gap. Based on electrical method and optical emission spectroscopy, it is found that gap voltage increases, while discharge current remains constant with increasing the gap width. Temperature and electron density of the discharge decrease with increasing the gap width.

## Introduction

In the past decades, atmospheric pressure glow discharge (APGD) has attracted increasing attention due to its operational flexibility afforded by the elimination of an expensive vacuum system. Spatially diffuse and non-equilibrium plasma can be generated in APGD^1, 2^, which has similar characteristics to the glow discharge at low pressure^3^. Therefore, it is desirable in diversified potential applications, such as biomedical treatment^4–6^, surface modification^7^, polymer deposition^8^, and nanoparticle formation^9^.

APGD is firstly reported in a dielectric-barrier discharge (DBD) geometry by Yokoyama *et al*.^10^. DBD-based APGD can be achieved by preventing glow-to-arc transition under strict conditions, such as a gas gap narrower than several millimeters^11^. Compared with DBD driven by an alternating current voltage, direct current (DC) glow discharge with naked electrodes is more simpler^12, 13^. Different electrode geometries and various working gases are employed to characterize DC glow discharges^1, 12, 14^. A thin cylindrical anode and a plate cathode are used to realize a DC APGD in air, hydrogen, helium and argon^1^. Using a DC micro-hollow cathode geometry, Zhu *et al*. have found that a normal glow discharge can transit into a self-pulsed one with decreasing current and oxygen content in helium^14^. Utilizing a rod anode and a spring cathode, Jiang *et al*. have reported a DC glow discharge in the flowing argon, whose cross-section increases from the anode to the cathode^12^.

Volume of the afore-mentioned APGDs is fairly small, especially in air environment. Scaling up APGD to large volume without compromising plasma uniformity remains a major challenge due to discharge instability (the glow-to-arc transition)^15–18^. Through cooling by a gas flow^12^, a planar DC APGD with a large volume is generated downstream of two rod electrodes^17^. Further investigations indicate that the planar discharge in a similar device consists of a series of moving filaments in an arched shape^19^. Additionally, employing array electrode is a solution to scale up APGD. Usually, only some discrete discharges are observed in the array electrode configuration^20, 21^. Mohamed *et al*. have reported a larger-volume air APGD through parallel operation of two independent discharges^22^, each of which has an anode and a plasma cathode originating from a micro-hollow cathode discharge. However, their electrode configuration is relatively complicated and the gap width is fairly narrow in the order of several millimeters.

In this paper, a simpler configuration is proposed to generate a planar air APGD with a relatively large volume through utilizing a rod array anode and a water cathode separated by several centimeters. With increasing the gap width between the rod anode and the cathode surface, the transition from three discrete APGDs to the planar APGD is investigated.

## Results

**Figure 1:**
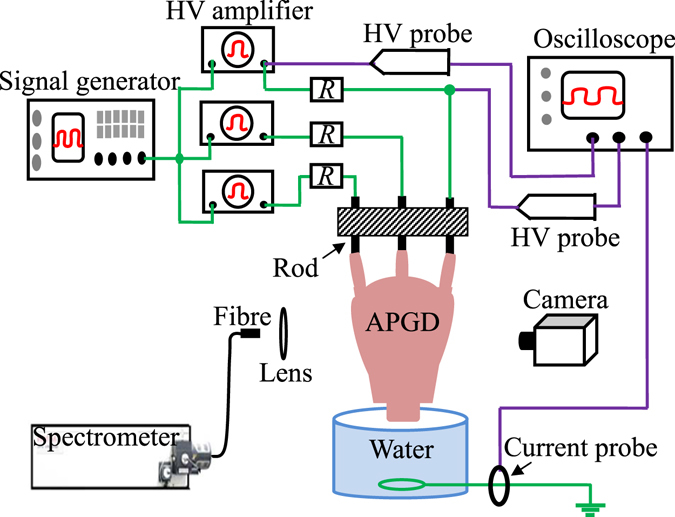
Schematic diagram of the experimental setup.

Figure 1 presents schematic diagram of the experimental setup. With increasing the amplifier output voltage to some extent (*V*_*o*_ is about 6.0 kV), atmospheric pressure air discharge initiates in a 2.5 mm gap between the rod anode and the water cathode. With the ignition of the discharge, the amplifier output voltage automatically decreases to about 2.6 kV. This phenomenon is explained in the discussion part. It should be emphasized that after the discharge ignition in a 2.5 mm gap, the amplifier output voltage (about 2.6 kV) keeps almost constant even if the amplifier input voltage is changed. It varies only with changing the gap width.

**Figure 2:**
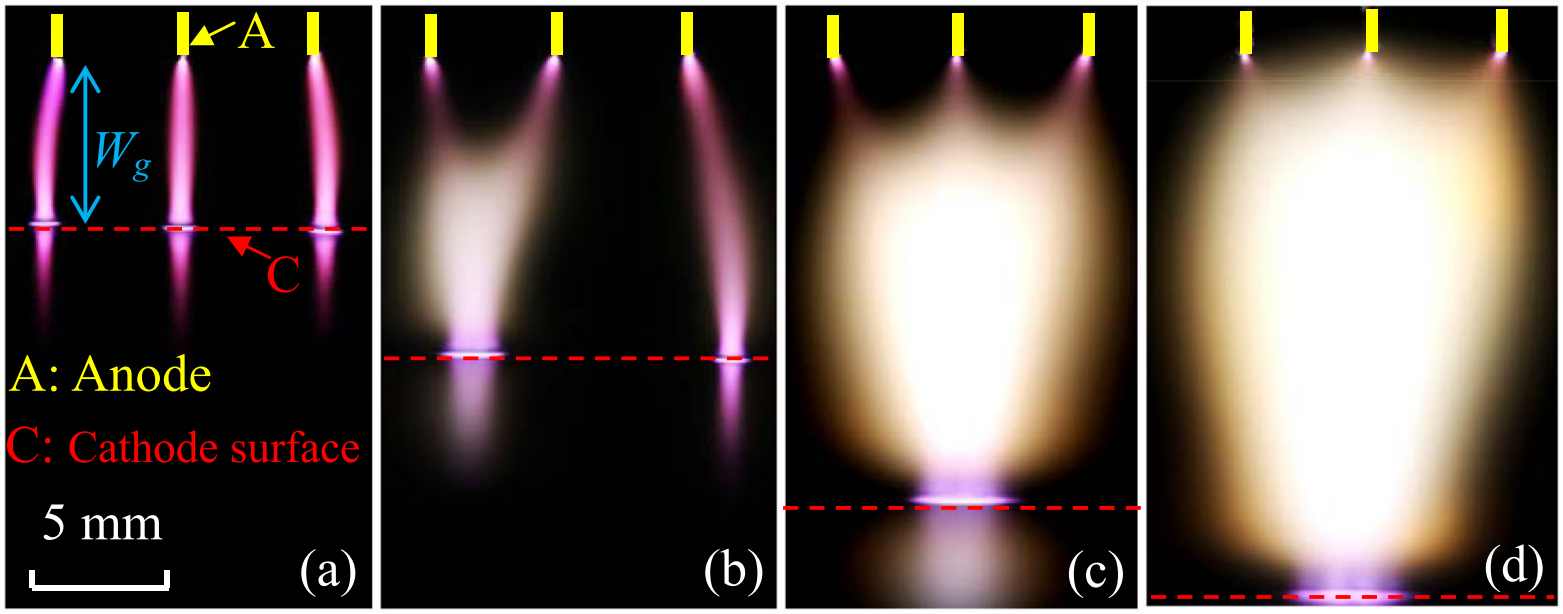
Discharge images under different gap widths: (**a**) 6 mm, (**b**) 11 mm, (**c**) 17 mm, (**d**) 20 mm. The exposure time is 125 ms.

With a narrower gap width (*W*_*g*_), three discrete discharges in a cylindrical shape bridge the anode (rod ends) and the cathode (water surface), as shown in Fig. 2(a). Light emission below the cathode surface results from light reflection on the water surface. On the water surface, there is repelling force between neighboring discharges because their touch points with the water surface deviate towards the two sides. With increasing *W*_*g*_, the middle parts of the discrete discharges bulge, resulting in a combination of two discharges, as shown in Fig. 2(b). Compared with Fig. 2(a), the deviation is much larger for the right discharge in Fig. 2(b), which indicates a stronger repelling force. Results also indicate that the middle discharge stochastically combines with the left discharge or the right one. With further increasing *W*_*g*_, the bulge continues until the discrete discharge joins the combined discharge. Consequently, a discharge in a planar shape (planar discharge, or laminar discharge) is formed, as shown in Fig. 2(c). Note that the discharges also merge in contact with water surface, which is different from the discharge reported by Mohamed *et al*.^22^. In their discharge, it is discrete in the vicinity of the cathode. Obviously, the planar discharge with a wide gap originates from the combination of the discrete discharges with a narrow gap. Volume of the planar discharge increases with further increasing *W*_*g*_, as shown in Fig. 2(d). It should be emphasized that the planar discharge will chaotically move on the water surface if the air gap is wider than 25 mm.

**Figure 3:**
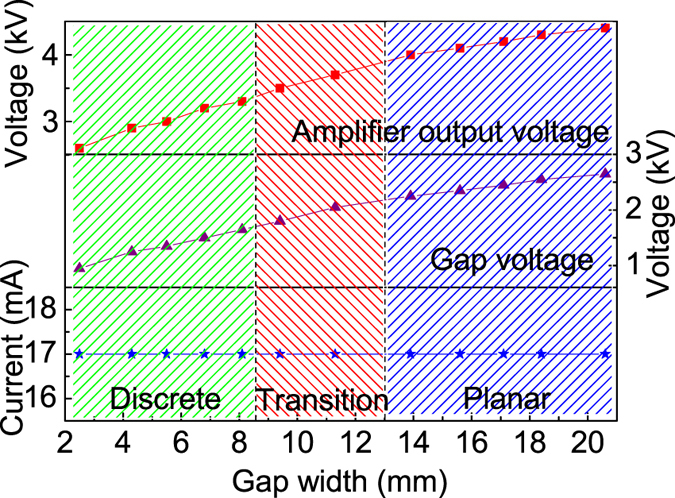
Amplifier output voltage, gap voltage and discharge current as functions of the gap width.

Figure 3 presents the amplifier output voltage (*V*_*o*_), the gap voltage (*V*_*g*_), and the discharge current as functions of the gap width under constant input voltage to the amplifiers (3 V). It can be found that *V*_*o*_ and *V*_*g*_ increase, while their slope rates decrease with increasing *W*_*g*_. Hence, it can be calculated that the applied electric field (*E*_*a*_) gradually decreases from 3.8 kV/cm to 1.3 kV/cm with increasing *W*_*g*_ from 2.5 mm to 20.6 mm. Additionally, every value of the current in Fig. 3 is averaged by ten measurement results. It can be found that the discharge current remains almost constant with increasing *W*_*g*_.

**Figure 4:**
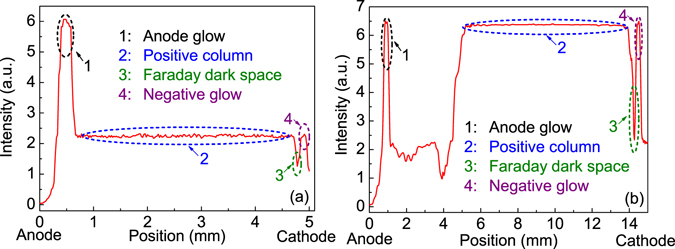
Spatially resolved light emission intensities along the middle discrete discharge with a gap width of 5 mm (**a**) and the symmetrical axis of the planar discharge with a gap width of 15 mm (**b**).

Figure 4 presents the spatially resolved light intensities obtained by the ICCD with an exposure time of 1 μs, which correspond to the middle rod-end at zero and the cathode surface at 5 mm and 15 mm, respectively. The middle discrete discharge with 5 mm *W*_*g*_ demonstrates an anode glow in the vicinity of the rod end, a positive column, a Faraday dark space, and a negative glow on the water surface, as shown in Fig. 4(a). For the planar discharge with 15 mm *W*_*g*_ (Fig. 4(b)), a dark region can be observed between the anode glow and the positive column besides the characteristic regions mentioned in Fig. 4(a). Existence of these characteristic regions verifies that both the discrete discharges and the planar discharge operate in the DC glow regime^12, 23^. It is noteworthy that the light intensity is not zero at the cathode surface, which also comes from light reflection of the discharge.

Considering the electrode configuration, the applied electric field (*E*_*a*_) has its maximal value at the rod end, and decreases with increasing distance from the anode. Therefore, the anode glow is most luminous at the rod end. Moreover, the negative glow originates from the formation of a positive-ion layer above the water surface^24^. There is repelling force between the positive-ion layers of the discrete discharges. Consequently, for the discrete discharges, the touch points with the water surface deviate towards the two sides in Fig. 2(a). After the combination of two discrete discharges, the repelling force is enhanced for the right discharge. Thus, its deviation becomes larger in Fig. 2(b). Additionally, electron diffusion can not be neglected along the radial direction of the positive column because of electron density gradient. As *E*_*a*_ decreases with increasing *W*_*g*_ as aforementioned, drift velocity for electrons decreases too. Therefore, drift time for electrons increases with increasing *W*_*g*_, resulting in a longer time for transverse diffusion of electrons. As a result, the positive columns of discrete discharges bulge with increasing *W*_*g*_, as shown in Fig. 2.

For the planar discharge, drift velocity of positive ions decreases after leaving the strong electric field region (the vicinity of the anode). Therefore, a layer of positive ions, similar to the positive-ion layer above the water surface, accumulates on the top of the positive column. Then, the electric field is reduced above it. Consequently, a dark region is observed between the anode glow and the positive column.

**Figure 5:**
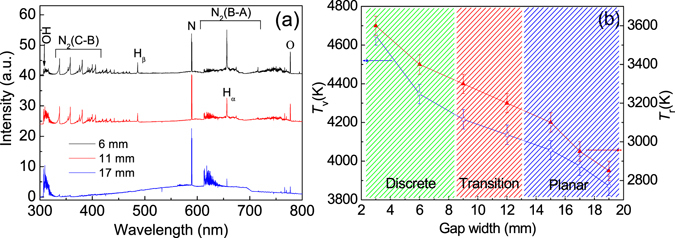
(**a**) Optical emission spectra from the positive columns of the discharges with a gap width of 6 mm (on the top), 11 mm (in the middle) and 17 mm (at the bottom), respectively. (**b**) Vibrational temperature and rotational temperature as functions of the gap width.

Figure 5(a) presents optical emission spectra from the discharges in Fig. 2(a,b and c), respectively. Typical spectral lines include OH transition ${A}^{2}{{\rm{\Sigma }}}^{+}(\nu =0,1)\to {X}^{2}{\rm{\Pi }}({\rm{\Delta }}v=0)$ at 308 nm, the second positive system of N_2_ transition ${C}^{3}{{\rm{\Pi }}}_{u}\to {B}^{3}{{\rm{\Pi }}}_{g}$ ^1^, the first positive system of N_2_ transition ${B}^{3}{{\rm{\Pi }}}_{g}\to {A}^{3}{{\rm{\Pi }}}_{u}$ ^25, 26^, OI transition $3{p}^{5}P\to 3{s}^{5}S$ at 777.4 nm, H_β_ transition 2*p* → 4*d* at 486.1 nm and H_α_ transition 2*p* → 3*d* at 656.3 nm. Table 1 presents significant spectral lines observed in the N_2_ second positive system. OH radicals and reactive oxygen species are beneficial for biomedical and material applications^27, 28^. In addition, an emission line from nitrogen atom is also observed at 589.1 nm with fairly strong intensity. From Fig. 5(a), it can be also found that the emissions from H_α_, H_β_, N_2_ (C-B) and OI are more intense for the discrete discharges, and OH radicals and N_2_ (B-A) have stronger intensity for the planar discharge. Moreover, a continuum is observed in the planar discharge spectrum, which can be attributed to the overlapping of abundant energy levels for the diatomic molecule (N_2_ (B-A)). Figure 5(b) presents the vibrational temperature (*T*_*v*_) and the rotational one (*T*_*r*_) obtained by fitting the second positive system of N_2_ and the OH radicals transition, respectively^29^. *T*_*r*_ nearly equals to the gas temperature of the discharge^29^. It is found from Fig. 5(b) that both *T*_*v*_ and *T*_*r*_ decrease with increasing *W*_*g*_. Moreover, *T*_*r*_ of the discrete discharge is consistent with that reported by Bruggeman^30^.

**Table 1:** Significant spectral lines observed in the N_2_ second positive system.

Electronic transition	Vibrational transition	Wavelength (nm)
*N*_2_ (*C-B*)	0–0	337.0
*N*_2_ (*C-B*)	1–2	353.6
*N*_2_ (*C-B*)	0–1	357.6
*N*_2_ (*C-B*)	2–4	370.9
*N*_2_ (*C-B*)	1–3	375.4
*N*_2_ (*C-B*)	0–2	380.4
*N*_2_ (*C-B*)	2–5	394.1
*N*_2_ (*C-B*)	1–4	399.7
*N*_2_ (*C-B*)	0–3	405.8

**Figure 6:**
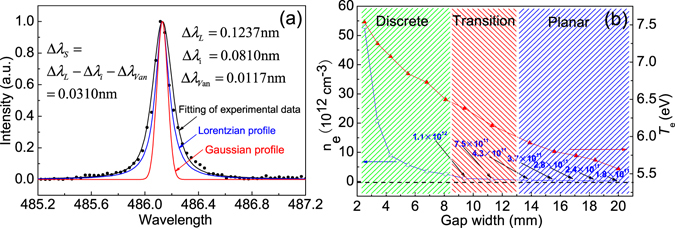
(**a**) Deconvolution process of H_β_ line (486.1 nm) emitted from the discrete discharges with a gap width of 2.5 mm. (**b**) Estimated electron density and electron temperature as functions of the gap width.

Stark broadening of H_β_ can be used to determine electron density (*n*_*e*_) higher than 10^13^ cm^−3^ ^31, 32^. Instrumental broadening at 491 nm line is measured by a mercury lamp with a slit width of 100 μm^28^. Figure 6(a) indicates a de-convolution procedure to obtain the Stark broadening of H_β_^33^. Its full width at half maximum (Δ*λ*_*S*_) is 0.0310 nm for the positive column of discrete discharges with 2.5 mm *W*_*g*_. Therefore, *n*_*e*_ is calculated by the relationship: ${n}_{e}={10}^{17}\times {({\rm{\Delta }}{\lambda }_{S}/4.8)}^{1.46808}$ to be 6.1 × 10^13^ cm^−3^. Additionally, ${n}_{e}=j/({E}_{p}{\mu }_{e}e)$, where *j* is current density, *E*_*p*_ is electric field in the positive column, *e* is electron charge. Electron mobility *μ*_*e*_ is 4.3 × 10^2^ cm^2^·(V·s)^−1^ at atmospheric pressure^34^. This estimation method is only valid for homogeneous plasmas, whose cross-section is relatively regular to reduce the measurement error^32^. By an extrapolation method^35^, voltage fall of the positive column is obtained to calculate *E*_*p*_. Through this method, *n*_*e*_ can be estimated, as shown in Fig. 6(b). It can be found that *n*_*e*_ decreases from 5.3 × 10^13^ cm^−3^ to 1.8 × 10^11^ cm^−3^ with increasing *W*_*g*_. The *n*_*e*_ value in the positive column is consistent with that reported by Mohamed *et al*.^22^. Apparently, for the discrete discharges with 2.5 mm *W*_*g*_, the calculated *n*_*e*_ (5.3 × 10^13^ cm^−3^) is in the same order with that obtained by the Stark broadening of H_β_ (6.1 × 10^13^ cm^−3^). As the ionization rate is fairly low for APGD^36^, it is valid to estimate the electron temperature (*T*_*e*_) of APGD by Einstein equation^37–39^. From Fig. 6(b), it is found that *T*_*e*_ decreases in the range from 7.5 eV to 5.5 eV with increasing *W*_*g*_. This phenomenon can be attributed to the decreasing applied electric field with increasing *W*_*g*_.

## Discussion

**Figure 7:**
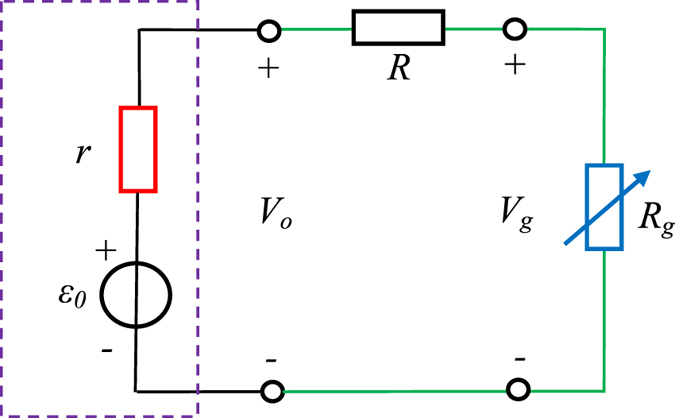
Equivalent circuit of the discharge.

Considering the internal resistance (*r*) of the voltage amplifier, the equivalent circuit of the discharge circuit is shown in Fig. 7. Here, *ε*_*0*_ is the electromotive force of the amplifier, *R* and *R*_*g*_ are the shunt resistance and the gap resistance, respectively. Obviously, *V*_*o*_ equals to *ε*_*0*_ because *R*_*g*_ is infinite before the discharge ignition, which corresponds to a broken circuit. After the discharge ignition, *R*_*g*_ significantly decreases to a small value. Apparently, ${V}_{{\rm{o}}}=\frac{R+{R}_{{\rm{g}}}}{R+{R}_{g}+r}{\varepsilon }_{0}$ and ${V}_{{\rm{g}}}={V}_{{\rm{o}}}-\frac{R}{R+{R}_{g}+r}{\varepsilon }_{0}$ in this case. Therefore, *V*_*o*_ has a significant drop after the discharge ignition. Moreover, *V*_*g*_ is lower than *V*_*o*_.

Effects of the shunt resistor and cathode material are investigated on the discharge behavior. The discharge still can be ignited when the shunt resistor is decreased to 10 kΩ from 100 kΩ. The current (about 19 mA) is closer to the upper limit of the amplifiers (20 mA). However, the discharge quenches in about 15 mm gap, which is narrower than that (about 25 mm) with 100 kΩ shunt resistor. Thereafter, reducing the shunt resistor takes disadvantage of generating a large-volume planar discharge. Investigation also indicates that the current decreases to about 9 mA when the shunt resistor is increased to 500 kΩ. For 500 kΩ resistor, the planar discharge becomes non-uniform observed by naked eyes. Moreover, maximal volume of the planar discharge also decreases with increasing the resistance. Consequently, the shunt resistor is fixed at 100 kΩ in order to generate the planar discharge with a fairly large volume. Additionally, the water cathode is replaced by a copper plate. Result indicates that a planar glow discharge can also be generated after the combination of three discrete discharges with increasing *W*_*g*_. When *W*_*g*_ is increased to about 10 mm, the planar discharge becomes unstable and begins to move chaotically on the plate. Finally, it quenches with the gap wider than about 12 mm. As liquid electrode has certain cooling effect to inhibit discharge instability (glow-to-arc transition)^40^, the planar discharge with a wider gap (about 20 mm) can be generated through using the water cathode, as shown in Fig. 2(d). Therefore, compared with the metal-plate cathode, it is more desirable to generate large-volume glow discharge with the water cathode.

As the glow discharge is generated in atmospheric pressure air, mean free path of electrons ($\overline{{\lambda }_{e}}$) keeps almost constant. Collision frequency between electrons and gas molecules (*v*) can be calculated by the formula: $\nu ={n}_{e}\frac{\overline{{\upsilon }_{{\rm{e}}}}}{\overline{{\lambda }_{{\rm{e}}}}}$. Due to the decrement of electron temperature (Fig. 6(b)), averaged velocity of electron ($\overline{{\upsilon }_{{\rm{e}}}}$) decreases with increasing *W*_*g*_. Moreover, electron density (*n*_*e*_) also decreases with increasing *W*_*g*_ (Fig. 6(b)). Obviously, collision frequency *v* decreases with increasing *W*_*g*_. Therefore, fewer collisions happen between electrons and gas molecules with increasing *W*_*g*_. Moreover, less efficient energy transfers from electron to heavy particles because of the decrement of electron temperature with increasing *W*_*g*_. Consequently, the gas temperature reduces with increasing *W*_*g*_ (Fig. 5(b)). Apparently, the decrement in gas temperature will restrain thermal instability, which is a main cause for the glow-to-arc transition^41^. Therefore, decreasing the gas temperature by widening the air gap promotes the generation of the planar DC glow discharge with a fairly large volume.

## Methods

A schematic diagram of the experimental setup is shown in Fig. 1. Three tungsten rods with a diameter of 1 mm and a length of 5 cm are placed in a line with 5 mm distance. The rod array is placed above a liquid reservoir with a volume of 1.4 L, in which tap water is filled to be used as a liquid electrode. Conductivity of the water electrode is about 1100 μS · cm^−1^. The water electrode is grounded through a stainless-steel ring located at its bottom. A signal generator (Tektronix AFG3021, maximum: 5 V) is used to produce a DC voltage of 3 V, which is simultaneously transmitted into three independent voltage amplifiers (Trek 20/20C-HS). Their high-voltage outputs are electrically connected to three rod electrodes via shunt resistors (*R* = 100 kΩ for each), respectively. Amplifier output voltage (*V*_*o*_) and gap voltage between the anodic rod and the cathode (*V*_*g*_) are detected by two high voltage probes (Tektronix P6015A), respectively. The current in the discharge circuit is detected by a current probe (Tektronix TCPA300). The voltages and the current are monitored simultaneously by an oscilloscope (Tektronix DPO4104). Discharge images are recorded by a digital camera (Canon EOS 7D) with a macro lens (Canon EF-S 60 mm f/2.8 USM). It is about 30 cm from the discharge to the lens. An intensified charge-coupled device (ICCD) (Andor DH334T) is also used to obtained the light emission intensity. A spectrometer (Acton SP2750) equipped with a CCD (Pixis 400) is used to collect the optical emission spectrum from the discharge in the range from 300–800 nm.
